# SOCS3 and IL-6 mRNA levels in PBMC enhance the early prediction for patients with acute-on-chronic hepatitis B liver failure receiving glucocorticoid therapy

**DOI:** 10.3389/fcimb.2025.1571443

**Published:** 2025-06-05

**Authors:** Ji-Hui Li, Jing Wang, Han-Xu Zhu, Feng Zhang, Yu-Na Tang, Jing-Wei Wang, Yu-Chen Fan, Hui-Hui Liu, Kai Wang

**Affiliations:** ^1^ Department of Hepatology, Qilu Hospital of Shandong University, Jinan, Shandong, China; ^2^ Department of Hepatology, Qilu Hospital of Shandong University (Qingdao), Qingdao, Shandong, China; ^3^ Institute of Hepatology, Shandong University, Jinan, China

**Keywords:** acute-on-chronic hepatitis B liver failure, glucocorticoid, prognosis prediction, SOCS3, IL-6

## Abstract

**Introduction:**

Early prediction is essential in hepatitis B virus-related acute-on-chronic liver failure (ACHBLF) patients receiving glucocorticoid therapy. Interleukin (IL)-6 is the main factor of cytokine storm that occurs in ACHBLF. IL-6 and suppressors of cytokine signaling 3 (SOCS3) may be associated with the prognosis of patients. Peripheral blood mononuclear cells (PBMCs), which are related to the immune response and inflammation of patients, are often used as materials for searching for biomarkers to predict ACHBLF. We aimed to determine serum cytokines levels and to evaluate the prediction value of SOCS3 and IL-6 mRNA levels in PBMC for ACHBLF patients with glucocorticoid therapy.

**Methods:**

Eighty-five patients with ACHBLF, fifty with pre-ACHBLF, fifty with CHB and thirty healthy controls (HCs) were enrolled. ACHBLF and pre-ACHBLF patients (n=135) received standard medical treatments (SMT) and 93 of them treated with glucocorticoids (SMT+GC). Serum cytokines levels, SOCS3 and IL-6 mRNA levels in peripheral blood mononuclear cells (PBMCs) were quantified.

**Results:**

Serum IL-6, IL-1ß, IL-18 and TNF-α levels of non-survivors with glucocorticoid therapy were significantly higher than that of survivors. Serum IL-10 levels in the survivors with glucocorticoid therapy were significantly increased. The analysis of variable importance shows that IL-6 ranks first. SOCS3 and IL-6 mRNA levels in PBMCs were significantly higher in ACHBLF patients than those in pre-ACHBLF patients (p=0.0158; p=0.0421). Non-survivors showed significantly lower SOCS3 levels (p=0.0084) and higher IL-6 levels (p<0 .0001) than survivors in ACHBLF patients. Non-survivors in patients receiving GC therapy had significantly lower SOCS3 levels (p=0.0002) and higher IL-6 levels (p<0 .0001) than survivors.SOCS3 and IL-6 mRNA levels were predictors for 90-day prognosis of patients receiving GC therapy (p=0.003; p=0.002). AUC of MELD score+SOCS3+IL-6 was 0.887 (95%CI, 0.818-0.955) and the optimal cut-off value was 0.29, with sensitivity of 93.3%, specificity of 74.6%. Patients receiving GC therapy with MELD score+SOCS3+IL-6=0.29 had a higher risk of poor prognosis (p<0 .0001). Survival rate in the SMT+GC group was higher than that in the SMT group (p=0.0350).

**Discussion:**

Glucocorticoid treatment could reduce the mortality of ACHBLF. SOCS3 and IL-6 mRNA combined with MELD score could be used to predict for ACHBLF patients receiving glucocorticoid therapy.

## Introduction

Acute-on-chronic liver failure (ACLF) is a distinct clinical syndrome in which patients with chronic liver disease, with or without cirrhosis, develop hepatic and extrahepatic organ failure, which mechanisms is still unclear but very likely involves inflammation (Moreau et al., 2021). In China, hepatitis B virus (HBV) infection is the main cause of ACLF, which is referred to as acute-on-chronic hepatitis B liver failure (ACHBLF) (Sarin et al., 2014). One important cause for the occurrence of ACHBLF in chronic hepatitis B (CHB) patients is HBV reactivation. This disease has a high short-term mortality and liver transplantation remains the definitive treatment until now (Sarin and Choudhury, 2016). However, there are still many challenges in the application of liver transplantation, such as organ shortage, complications of long-term immunosuppression and chronic immune-mediated injury to the liver (Jadlowiec and Taner, 2016). Apart from the aforementioned challenges, one of the most important challenge in selecting liver transplantation for ACLF patients is the cost involve. Furthermore, acute-on-chronic hepatitis B pre-liver failure (pre-ACHBLF), as the previous stage of ACHBLF, if not treated promptly and effectively, is more likely to progress to ACHBLF and the prognosis of patients will be worse. Early and effective management of ACHBLF and pre-ACHBLF is essential for reducing patients’ mortality. Therefore, new early predictive methods and highly effective therapy is important for improving the prognosis of ACHBLF and pre-ACHBLF patients.

The acute insult, inflammatory injury, immune deregulation, systemic inflammatory response syndrome (SIRS), compensatory anti-inflammatory response syndrome (CARS) and the regenerative response are associated with occurrence and progression of ACHBLF (Sarin and Choudhury, 2016). Immune dysfunction is central to the pathogenesis and outcome of ACHBLF, with systemic inflammatory responses leading to organ failure and mortality (Casulleras et al., 2020). For the treatment of ACHBLF patients, it is important to effectively suppress the immune response, anti-inflammatory and antiviral effects. Glucocorticoids has pharmacological effects that influence metabolic, anti-inflammatory, immunosuppressive, and cognitive signaling processes, which are widely used for the treatment of asthma, Crohn’s disease, and rheumatoid arthritis and other chronic conditions (Scherholz et al., 2019). Previous studies have shown that corticosteroid combined with nucleoside analogues have significant efficacy and sufficient virological effect in the early stage of severe acute exacerbation of CHB (Yasui et al., 2015). Therefore, the application of glucocorticoid therapy for ACHBLF has better clinical benefits. However, there are some side effects in glucocorticoids treatment (Schäcke et al., 2002). In addition, some patients who have no response to glucocorticoid treatment have poor prognosis. Few studies have been conducted to predict the prognosis of ACHBLF and pre-ACHBLF patients treated with glucocorticoids and it is difficult to predict the efficacy of glucocorticoids treatment. Therefore, there is an urgent need for early prediction of ACHBLF and pre-ACHBLF patients receiving glucocorticoid therapy.

Disruption of the homeostasis of the immune system can upset the balance between pro- and anti-inflammatory effects, leading to the release of a large amount of cytokines, namely cytokine storm, and causing systemic damage, multiple organ failure or death (Jarczak and Nierhaus, 2022). Infection-related cytokine storm, such as that caused by viral infection, can induce profound cytokine storm. The virus impairs the inflammatory regulation mechanism of patients, preventing them from effectively eliminating inflammation (Fajgenbaum and June, 2020). If HBV infection in the liver is not effectively controlled, it may lead to liver failure. Cytokine storm is one of the pathological mechanisms of ACHBLF. The excessive systemic inflammatory response caused by impaired immune function makes patients more susceptible to virus infections, thereby leading to organ dysfunction and increased mortality (Casulleras et al., 2020). Interleukin (IL)-6, as the core molecule of cytokine storm, and its Janus kinase (JAK)/signal transducer and activator of transcription (STAT)3 signaling pathway play an important role in its occurrence and development (Jones and Jenkins, 2018). Suppressors of cytokine signaling (SOCS)3 acts as a negative regulator of IL-6-JAK/STAT3 signaling pathway, which is involved in regulating related cytokines in infectious and inflammatory diseases (Gao et al., 2018). In addition, circulating immune cells (peripheral blood mononuclear cells, PBMCs) are distributed throughout various organs via the blood circulation and associated with the immune responses and inflammation regulation of patients, which are often used as materials for searching biomarkers that predict the development of ACHBLF (Li et al., 2022; Hassan et al., 2024). Until now, our previous studies found that SOCS1 promoter methylation level and thymosin β4 (Tβ4) promoter methylation level in PBMC were good prediction biomarkers for the prognosis of ACHBLF patients received glucocorticoid treatment (Zhang et al., 2014; Wang et al., 2023). However, few studies have evaluated the role of SOCS3 and IL-6 mRNA levels in PBMC for early prediction of ACHBLF patients treated with glucocorticoids.

In this study, SOCS3 and IL-6 mRNA levels were evaluated in the peripheral blood mononuclear cells (PBMCs) of ACHBLF and pre-ACHBLF patients. Early prediction value based on SOCS3, IL-6 mRNA levels and MELD score for ACHBLF patients receiving glucocorticoid therapy were evaluated to guide disease treatment.

## Methods

### Patients selection and enrollment

Patients with ACHBLF (n=85), pre-ACHBLF (n=50), CHB (n=50) and healthy controls (HCs) (n=30) in Department of Hepatology, Qilu Hospital of Shandong University from November 2021 to December 2024 were enrolled in this study. ACHBLF patients were diagnosed according to consensus recommendations of the Asian Pacific association for the study of the liver (APASL) (Sarin et al., 2014). The diagnostic criteria for pre-ACHBLF was based on guideline for diagnosis and treatment of liver failure as follows: TBIL (85.5-170μmol/L) and PTA (40%-50%) (Liver Failure and Artificial Liver Group et al., 2019). CHB was determined based on Asian-Pacific clinical practice guidelines on the management of hepatitis B (Terrault et al., 2016).

Patients were excluded if they fulfilled one or more of the following criteria: HCC, autoimmune liver diseases, alcoholic hepatitis, hepatitis C virus (HCV), hepatitis E virus (HEV), hepatitis G virus (HGV), known decompensated cirrhosis prior to onset of acute hepatic insult, human immunodeficiency virus (HIV), any other type of immunodeficiency, antioxidant use, interferon therapy, age less than 18 years, pregnancy, used glucocorticoids during 6 months prior to this study, loss to follow-up.

The Medical Ethical Committee of Qilu Hospital of Shandong University approved this study according to the guidelines of the 1975 Declaration of Helsinki, with the ethical approval number “KYLL-202111-244-2” and informed consent was obtained from all patients.

### Patients treatment

All patients received standard medical treatments (SMT) during hospitalization, including bed rest, nutritional support therapy, oral nucleos (t) ide analogues (such as entecavir, tenofovir, etc.), symptomatic support treatment, etc. SMT was performed based on consensus recommendations of the Asian Pacific association for the study of the liver (APASL) (Sarin et al., 2014).

ACHBLF and pre-ACHBLF patients were divided into SMT group and additional glucocorticoid treatment (SMT+GC) group according to whether glucocorticoids were used. ACHBLF patients were divided into SMT group (n=27) and SMT+GC group (n=58); pre-ACHBLF patients were divided into SMT group (n=15) and SMT+GC group (n=35).

Glucocorticoid treatment was given to patients with prednisolone 0.75mg/(kg day) (mean 60mg/day) for 3 days. The treatment was changed to prednisolone 0.5mg/(kg day) (mean 40mg/day) for 3 days, and then reduced to prednisolone 0.25mg/(kg day) (mean 20mg/day) for 3 days for a total of 9 days. The dosage of prednisolone was reduced by 5-10mg every 4 days according to the improvement of liver function, and maintained at 10mg daily until 28 days (Zhang et al., 2014).

### Patients study and follow-up

Clinical laboratory parameters and peripheral blood samples of ACHBLF and pre-ACHBLF patients were collected at 0 day after admission. The following Clinical laboratory parameters were measured using the operating procedures of the Department of Medicine Laboratory, Qilu Hospital, Shandong University: alanine aminotransferase (ALT), aspartate aminotransferase (AST), albumin (ALB), total bilirubin (TBIL), creatinine (Cr), blood urea nitrogen (BUN), prothrombin time activity (PTA), prothrombin time-international normalized ratio (INR), hepatitis B e antigen (HBeAg) and serum HBV-DNA. The model for end-stage liver disease (MELD) score was calculated by the formula. *MELD score=9.57×ln [creatinine (mg/dL)]+3.78×ln [bilirubin(mg/dL)]+11.2×ln (INR)+6.43×(etiology: 0 if cholestatic or alcoholic, 1 if due to other causes) (* Malinchoc et al., 2000).

At the end of the 90-day follow-up, the prognosis of ACHBLF and pre-ACHBLF patients were defined as non-survivors or survivors. In this study, the 90d prognosis of patients receiving glucocorticoid therapy was regarded as the effect of glucocorticoid therapy, and non-survivors receiving glucocorticoid therapy were considered as having no response to glucocorticoid treatment.

### Serum and PBMCs collection from peripheral venous blood

5mL peripheral venous blood was obtained from each patients in tubes containing ethylenediaminetetraacetic acid (EDTA). The collected peripheral blood was centrifuged. The serum was separated after centrifugation. Peripheral blood mononuclear cells (PBMCs) were isolated via Ficoll-Paque density gradient centrifugation. The serum was used for subsequent detection of cytokines. PBMCs were used immediately for subsequent experiments or preserved at −80°C until use.

### Total RNA extraction and reverse transcription

Total RNA was extracted from peripheral blood mononuclear cells (PBMCs) by TRIzol reagent (Invitrogen, Carlsbad, CA, USA). The concentration and purity of total RNA were determined using a DeNovix DS-11 + spectrophotometer. The extracted RNA was converted into complementary DNA (cDNA) by the frst-strand cDNA synthesis kit (Fermentas, Vilnius, Lithuania). The obtained cDNA was used immediately for subsequent experiments or preserved at −80°C until use.

### Measurement of SOCS3 and IL-6 mRNA expression by quantitative real-time polymerase chain reaction

SOCS3 and IL-6 mRNA expression was measured by RT-qPCR with ACTB serving as the internal control. The SOCS3, IL-6 and ACTB mRNA primer sequences are showed in **Table 1**. The reaction was performed under the following conditions: initial denaturation at 95°C for 10 min, then 40 cycles of denaturation at 95°C for 5 s, annealing at 58°C for 30 s and extension at 72°C for 30 s. The SOCS3 and IL-6 mRNA expression was calculated by the 2^−ΔΔCT^ method.

**Table 1:** Primer sequences of the genes used by RT-qPCR.

Gene	Forward primer sequence (5’-3’)	Reverse primer sequence (5’-3’)
SOCS3	TCTGTCGGAAGACCGTCAAC	CCTTAAAGCGGGGCATCGTA
IL-6	ACTCACCTCTTCAGAACGAATTG	CCATCTTTGGAAGGTTCAGGTTG
ACTB	ATGGGTCAGAAGGATTCCTATGTG	CTTCATGAGGTAGTCAGTCAGGTC

### Measurement of serum cytokines by enzyme-linked immunosorbent assay

The levels of serum cytokines were determined by using the Human Immunoassay Valukine ELISA Kits for IL-6, IL-10, TNF-α, IL-1β, and IL-10 (Shanghai Leighton Biotechnology Co., Ltd.). These kits detect the content in serum by the competitive method. According to the manufacturer’s protocol, the absorbance was measured at 450 nm.

### Statistical analysis

Statistical analyses were performed using the following software: SPSS (version 27.0; IBM SPSS Inc., Chicago, IL), GraphPad (GraphPad Prism version 9.5.1) and R (version 4.0.1). Categorical variables were indicated as numbers (proportions). Normally distributed variables were expressed as means ± standard deviations, whereas non-normally distributed variables were expressed as medians with interquartile ranges (P25-P75). Categorical variables were compared using the chi-square test. Mann-Whitney U test or Kruskal-Wallis test was used for comparison between two groups or more. The importance of the cytokine indicators was analyzed and ranked by the random forest method. Univariate and multivariate logistic regression analysis were used to select the predictive factors affecting the prognosis. Any p values less than 0.05 were considered statistically significant.

## Results

### Baseline characteristics of the enrolled patients and healthy controls

**Figure 1:**
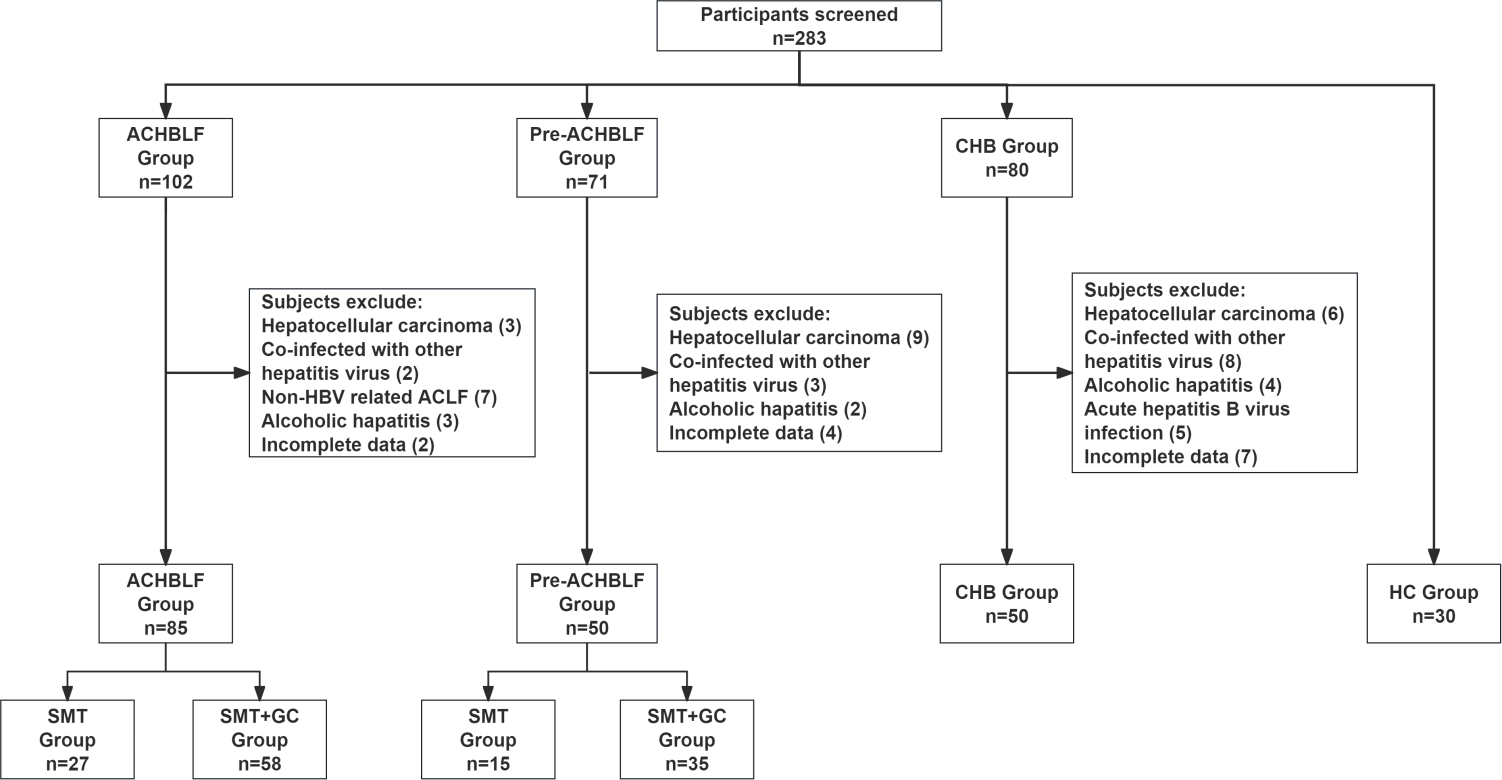
Flow chart of patient enrollment and grouping.

The process for patients and healthy controls selection and enrollment was shown in **Figure 1**. In this study, a total of ACHBLF patients (n=85), pre-ACHBLF patients (n=50), CHB (n=50) and HCs (n=30) were finally enrolled. The baseline characteristics of the enrolled patients on 0 day of admission and healthy controls (HCs) were presented in **Table 2**. Compared with pre-ACHBLF, CHB and HC groups, ALT, AST, TBiL, PTA and INR in the ACHBLF group were significantly increased. The MELD score of ACHBLF patients was significantly higher than that of pre-ACHBLF patients.

**Table 2:** Baseline characteristics of the enrolled patients and healthy controls.

Variable	ACHBLF patients (n=85)	pre-ACHBLF patients (n=50)	CHB patients (n=50)	HCs group (n=30)
Age (years)	50.00 (41.00-59.00)	49.00 (37.25-57.50)	50.00 (43.25-55.50)	48.00 (43.25-51.75)
Gender (male,%)	59 (69.41)	38 (76.00)	35 (70.00)	15 ( 50.00)
HBeAg positive (n,%)	52 (61.18)	30 (60.00)	28 (56.00)	NA
HBV DNA (log10 IU/ml)	3.75 (2.72-5.31)	3.59 (2.61-5.18)	3.59 (2.76-5.88)	NA
ALT (U/L)	195.00 (153.00-278.00)	130.00 (85.75- 252.75) ** ^b^	31.00 (17.25-42.75) *** ^b^	26.00 (20.00-30.75) *** ^b^
AST (U/L)	175.00 (144.00-272.00)	126.50 (87.75-176.50) *** ^b^	27.00 (20.00-32.00) *** ^b^	19.00 (14.25-26.00) *** ^b^
ALB (g/L)	32.60 (29.80-36.60)	32.95 (30.05-35.90)	48.15 (46.42-50.18) *** ^b^	40.15 (37.90-45.90) *** ^b^
TBiL (μmol/L)	274.40 (214.10-379.30)	126.40 (105.95-144.95) *** ^b^	11.00 (8.85-14.57) *** ^b^	9.35 (6.55-11.20) *** ^b^
Cr (μmol/L)	55.00 (46.00-69.00)	58.50 (46.25-72.25)	64.50 (58.00-74.75) *** ^b^	72.00 (64.25-77.75) *** ^b^
BUN (mmol/L)	5.40 (3.90-7.70)	4.20 (3.20-6.38) * ^b^	3.94 (2.98-4.36) *** ^b^	3.42 (2.80-3.91) *** ^b^
PTA	35.00 (30.00-44.00)	46.00 (43.00-47.00) *** ^b^	98.00 (84.50-118.75) *** ^b^	120.50 (104.25-129.75) *** ^b^
INR	1.82 (1.64-2.36)	1.69 (1.52-1.86)	0.95 (0.86-1.03) *** ^b^	0.98 (0.94-1.10) *** ^b^
MELD score	19.46 (17.32-23.08)	15.85 (12.94-19.35) *** ^b^	NA	NA

Quantitative variables were expressed as the means±SD or the median (centile 25; centile 75). Categorical variables were expressed as number (%).

*p<0.05, **p<0.01, ***p<0.001 and ****p<0.0001 for comparisons between pre-ACHBLF/CHB/NC and ACHBLF group. a unpaired t test. b Mann–Whitney U test.

ACHBLF, acute-on-chronic hepatitis B liver failure; CHB, chronic hepatitis B; HC, healthy controls; HBeAg, hepatitis B virus e antigen; HBV, hepatitis B virus; ALT, alanine aminotransferase; AST, aspartate transaminase; ALB, Albumin; TBiL, total bilirubin; Cr, creatinine; BUN, blood urea nitrogen, PTA, prothrombin activityprothrombin time activity; INR, international normalized ratio; MELD, model for end-stage liver disease; NA, not applicable.

### Serum cytokines levels in patients receiving glucocorticoid therapy with different 90d prognosis

**Figure 2:**
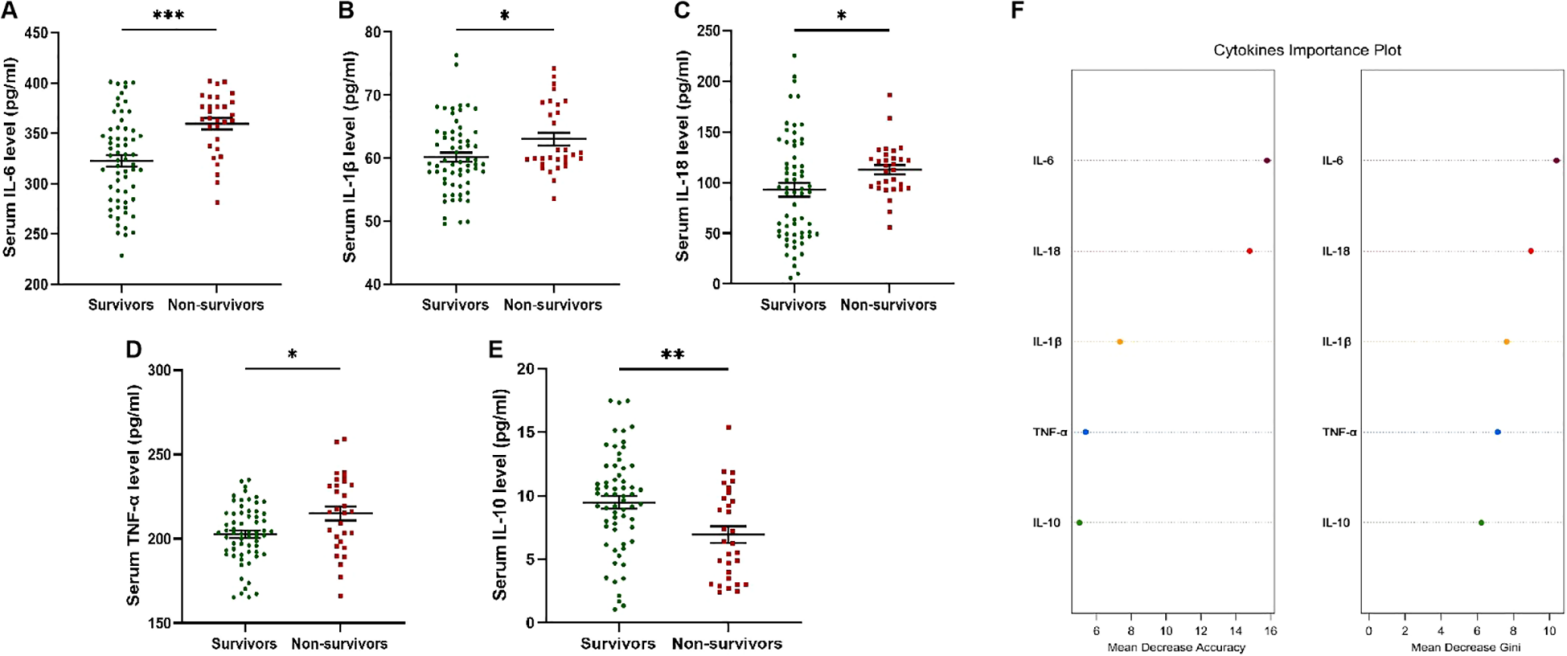
Serum cytokines levels in patients receiving glucocorticoid therapy with different 90d prognosis. **(A)** Serum IL-6 levels of non-survivors were significantly higher than that of survivors. **(B)** Serum IL-1β levels of non-survivors were significantly higher than that of survivors. **(C)** Serum IL-18 levels of non-survivors were significantly higher than that of survivors. **(D)** Serum TNF-α levels of non-survivors were significantly higher than that of survivors. **(E)** Serum IL-10 levels of survivors were significantly higher than that of non-survivors. **(F)** Cytokines important plot was drawn to evaluate the importance. * p<0.05; ** p<0.01; *** p<0.001.

Based on the 90-day prognosis, patients receiving glucocorticoid therapy (n=93) were divided into survivors (n=63) and non-survivors (n=30). Serum IL-6, IL-1β, IL-18 and TNF-α levels of non-survivors were significantly higher than that of survivors (*p*=0.0001, 0.0197, 0.0230, 0.0112) (**Figures 2A–D**). Compared with non-survivors, serum IL-10 levels in the survivors were significantly increased (*p*=0.0045) (**Figure 2E**). According to the analysis of the importance of serum cytokine levels for the 90-day prognosis of patients, it was found that the serum IL-6 level was of relatively high importance (**Figure 2F**).

### IL-6 and SOCS3 mRNA levels in different groups and their correlation with clinical characteristics

By analyzing the importance of serum cytokine levels, it was found that serum IL-6 levels increased and played an important role in predicting the prognosis of patients. Peripheral blood mononuclear cells (PBMCs) reflects the immune and inflammatory status of patients. Therefore, the determination of IL-6 mRNA level and its main inhibitory factor SOCS3 mRNA level in PBMCs can be used for biomarkers assessment.

**Figure 3:**
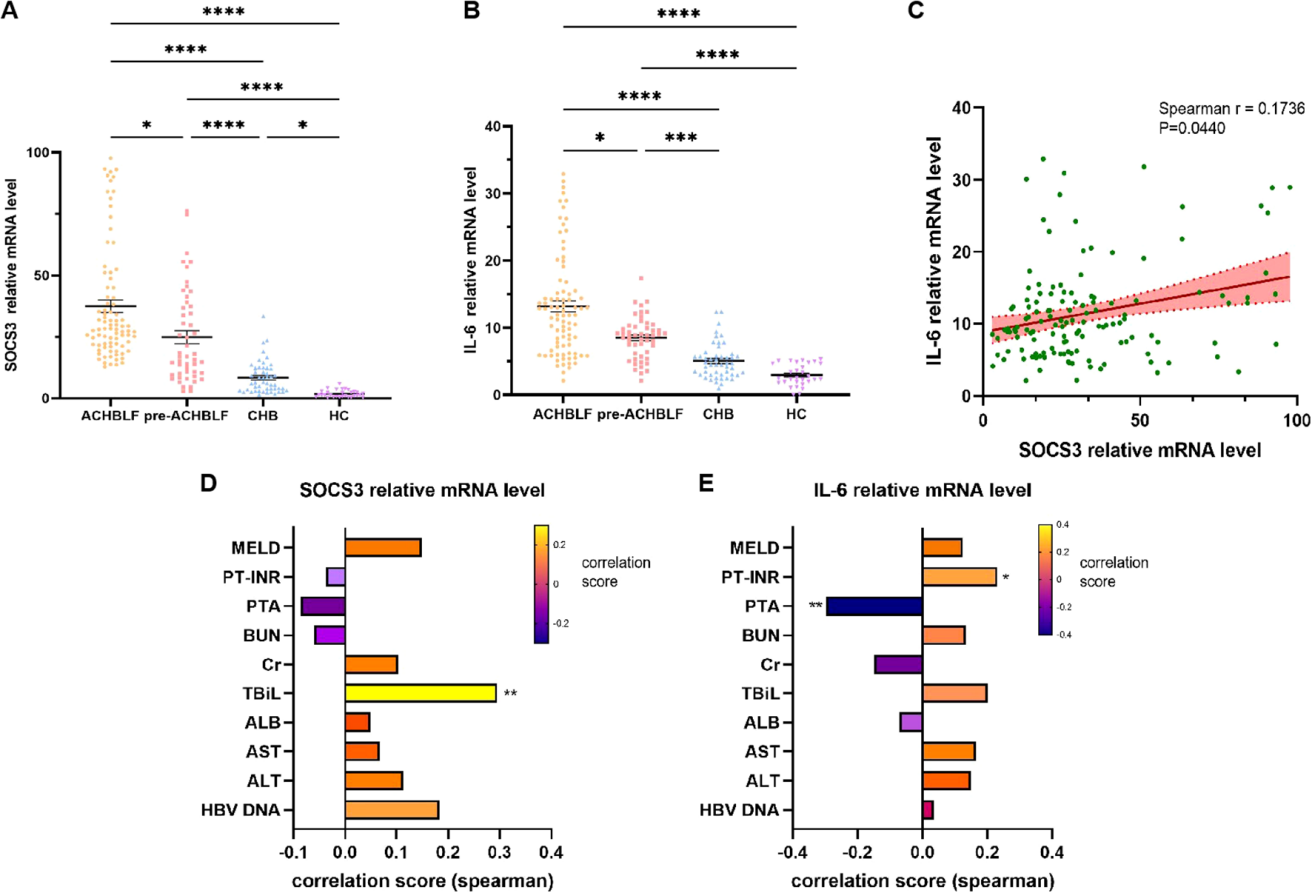
SOCS3 and IL-6 mRNA levels in different groups and correlation with clinical characteristics. **(A)** SOCS3 mRNA levels were compared between ACHBLF, pre-ACHBLF, CHB and HCs groups. **(B)** IL-6 mRNA levels were compared between ACHBLF, pre-ACHBLF, CHB and HCs groups. **(C)** SOCS3 mRNA levels were significantly positively correlated with IL-6 mRNA levels. **(D)** Correlation between SOCS3 mRNA levels and clinical characteristics in ACHBLF and pre-ACHBLF patients. **(E)** Correlation between IL-6 mRNA levels and clinical characteristics in ACHBLF and pre-ACHBLF patients. * p<0.05; ** p<0.01; *** p<0.001; ****p<0.0001.

As shown in **Figure 3A**, compared with pre-ACHBLF, CHB and HC groups, SOCS3 mRNA levels were significantly higher in ACHBLF group (*p*=0.0158, <0.0001, <0.0001). SOCS3 mRNA levels were significantly higher for CHB patients (n=50, 6.25[3.17-12.30]) than for HCs (n=30, 1.36[0.67-2.74]) (*p*=0.0120). In addition, SOCS3 mRNA levels of pre-ACHBLF group were significantly increased than that of CHB and HC groups (*p*<0.0001, <0.0001). In **Figure 3B**, IL-6 mRNA levels were significantly higher in ACHBLF group than that of pre-ACHBLF, CHB and HC groups (*p=*0.0421, <0.0001, <0.0001). Compared with pre-ACHBLF group, IL-6 mRNA levels in CHB and HC groups were significantly lower (*p*=0.0002, <0.0001). **Figure 3C** showed that SOCS3 mRNA levels were significantly positively correlated with IL-6 mRNA levels (Spearman’s r=0.1736, *p*=0.0440). SOCS3 mRNA levels were significantly correlated with TBiL (Spearman’s r=0.2952, *p*=0.0041) (**Figure 3D**). In addition, IL-6 mRNA levels were significantly correlated with PTA (Spearman’s r=-0.2979, *p*=0.0037) and INR (Spearman’s r=0.2304, *p*=0.0263) (**Figure 3E**).

### General characteristics of ACHBLF and pre-ACHBLF patients with different treatment and different 90d prognosis

As shown in **Table 3**, ACHBLF patients (n=85) were divided into SMT group (n=27) and SMT+GC group (n=58). Cr was significantly higher in SMT group (*p*=0.0090). In addition, pre-ACHBLF patients (n=50) were divided into SMT group (n=15) and SMT+GC group (n=35). MELD score was significantly higher in SMT group (*p*=0.0350). In addition, different treatment groups were further divided according to 90-day prognosis. General characteristics of patients with different treatment in different 90d prognosis was shown in **Table 4**. MELD score and INR were significantly higher in non-survivors of all ACHBLF treatment groups. PTA was significantly higher in survivors of ACHBLF SMT+GC group. In addition, ALT was significantly higher in survivors of pre-ACHBLF SMT group. BUN was significantly higher in non-survivors of pre-ACHBLF SMT+GC group.

**Table 3:** General characteristics of SMT group and SMT+GC group of ACHBLF and pre-ACHBLF patients.

Variable	ACHBLF patients (n=85)			pre-ACHBLF patients (n=50)		P value
	SMT group (n=27)	SMT+GC group (n=58)	P value	SMT group (n=15)	SMT+GC group (n=35)	
Age (years)	50.00 (42.00-63.50)	49.50 (41.00-58.00)	0.992 ^b^	49.00 (42.00-56.00)	50.00 (36.50-58.00)	0.907 ^b^
Gender (male,%)	20 (74.07)	39 (67.24)	0.524 ^b^	12 (80.00)	26 (74.29)	0.665 ^b^
HBeAg positive (n,%)	15 (55.55)	37 (63.79)	0.468 ^b^	12 (80.00)	18 (51.43)	0.059 ^b^
HBV DNA (log10 IU/ml)	3.98 (2.68-5.45)	3.71 (2.72-4.92)	0.730 ^b^	3.25 (2.64-4.64)	3.61 (2.75-5.34)	0.759 ^b^
ALT (U/L)	194.00 (128.50-265.50)	196.00 (155.50-278.00)	0.512 ^b^	134.00 (93.00-342.00)	122.00 (80.00-248.00)	0.589 ^b^
AST (U/L)	173.00 (142.50-271.00)	176.50 (146.50-270.75)	0.861 ^b^	133.00 (101.00-218.50)	125.00 (89.50-168.50)	0.446 ^b^
ALB (g/L)	32.80 (30.50-36.85)	32.45 (29.50-35.62)	0.439 ^b^	33.40 (31.60-36.30)	32.20 (28.45-35.25)	0.182 ^b^
TBiL (μmol/L)	301.80 (202.90-411.55)	273.55 (224.85-357.88)	0.692 ^b^	122.30 (110.90-137.35)	128.50 (100.55-146.20)	0.992 ^b^
Cr (μmol/L)	66.00 (49.50-77.00)	52.50 (44.25-62.00)	0.009 ^b^	58.00 (49.50-75.00)	59.00 (41.50-67.50)	0.299 ^b^
BUN (mmol/L)	5.40 (4.05-8.60)	5.42 (3.82-6.47)	0.406 ^b^	3.60 (3.30-6.95)	4.30 (3.20-6.00)	1.000 ^b^
PTA	36.00 (30.50-45.00)	35.00 (29.25-43.75)	0.723 ^b^	46.00 (43.50-47.00)	46.00 (43.00-47.50)	0.940 ^b^
INR	1.74 (1.58-2.22)	1.96 (1.67-2.39)	0.222 ^b^	1.77 (1.51-2.24)	1.69 (1.52-1.83)	0.440 ^b^
MELD score	21.33 (18.47-23.99)	19.02 (16.97-22.32)	0.157 ^b^	17.65 (15.34-21.23)	14.47 (11.98-18.34)	0.035 ^b^

Quantitative variables were expressed as the means±SD or the median (centile 25; centile 75). Categorical variables were expressed as number (%).

a unpaired t test. b Mann–Whitney U test.

SMT, standard medical treatment; SMT+GC, standard medical treatment plus glucocorticoid; ACHBLF, acute-on-chronic hepatitis B liver failure; CHB, chronic hepatitis B; HC, healthy controls; HBeAg, hepatitis B virus e antigen; HBV, hepatitis B virus; ALT, alanine aminotransferase; AST, aspartate transaminase; ALB, Albumin; TBiL, total bilirubin; Cr, creatinine; BUN, blood urea nitrogen, PTA, prothrombin activityprothrombin time activity; INR, international normalized ratio; MELD, model for end-stage liver disease.

**Table 4:** General characteristics of SMT group and SMT+GC group with different 90-day prognosis.

Variable	ACHBLF patients (n=85)				pre-ACHBLF patients (n=50)			
	SMT group (n=27)		SMT+GC group (n=58)		SMT group (n=15)		SMT+GC group (n=35)	
	Survivors (n=15)	Non-survivors (n=12)	Survivors (n=39)	Non-survivors (n=19)	Survivors (n=7)	Non-survivors (n=8)	Survivors (n=24)	Non-survivors (n=11)
Age (years)	50.00 (43.00-63.50)	49.50 (42.25-57.50)	48.00 (40.50-58.00)	52.00 (48.00-57.50)	43.00 (37.00-50.50)	56.00 (47.50-59.00) * ^b^	50.50 (35.00-58.50)	48.00 (38.00-57.00)
Gender (male,%)	9 (60.00)	11 (91.67)	27 (69.23)	12 (63.16)	6 (85.71)	4 (50.00) * ^b^	18 (75.00)	8 (72.73)
HBeAg positive (n,%)	7 (46.67)	8 (66.67)	26 (66.67)	11 (57.89)	5 ( 71.43)	5 (62.50)	14 (58.33)	4 (36.36)
HBV DNA (log10 IU/ml)	3.98 (2.76-6.59)	3.42 (2.72-4.77)	3.68 (2.81-4.82)	4.23 (2.66-5.38)	3.46 (2.91-4.78)	3.07 (2.57-4.49)	3.60 (2.83-5.90)	3.61 (2.83-4.16)
ALT (U/L)	164.00 (124.50-265.50)	220.00 (162.50-274.50)	192.00 (149.50-253.00)	214.00 (167.00-278.00)	342.00 (132.75-591.50)	95.00 (81.00-133.00) * ^b^	126.50 (72.00-255.50)	103.00 (90.00-187.00)
AST (U/L)	169.00 (139.50-236.50)	191.50 (143.00-280.00)	178.00 (147.00-270.50)	175.00 (147.00-263.00)	218.50 (121.25-412.50)	124.00 (82.50-144.50)	122.50 (95.00-166.75)	133.00 (84.50-161.50)
ALB (g/L)	32.30 (30.30-36.55)	32.80 (31.05-40.67)	33.50 (29.55-36.25)	31.60 (29.60-34.05)	36.30 (33.38-37.93)	33.30 (31.60-33.35)	32.30 (28.58-35.05)	30.50 (28.65-37.00)
TBiL (μmol/L)	238.40 (186.80-384.00)	336.25 (248.07-444.30)	266.30 (218.20-374.45)	274.40 (235.35-325.20)	133.15 (122.78-141.88)	114.70 (106.75-121.55)	127.50 (99.25-146.10)	129.30 (109.00-150.55)
Cr (μmol/L)	53.00 (46.00-81.50)	69.50 (63.25-73.50)	51.00 (43.00-59.00)	56.00 (47.50-85.50)	57.00 (50.25-66.25)	61.00 (53.00-102.00)	60.50 (49.00- 71.50)	47.00 (36.00-59.50)
BUN (mmol/L)	5.10 (2.75-11.50)	6.90 (4.90-7.92)	5.00 (3.70-6.25)	6.10 (4.23-8.55)	3.45 (2.89-4.40)	6.30 (3.65-8.10)	4.02 (3.18- 5.62)	6.40 (4.25-8.55) * ^b^
PTA	36.00 (31.00-45.00)	34.50 (30.00-41.50)	36.00 (32.50-44.50)	30.00 (26.50-41.50) * ^b^	46.50 (43.75-47.00)	45.00 (44.00-47.00)	47.00 (43.00-48.00)	46.00 (42.50-47.00)
INR	1.66 (1.51-1.77)	2.09 (1.73-2.38) * ^b^	1.78 (1.59-2.16)	2.39 (1.81-2.72) ** ^b^	1.60 (1.52-2.02)	1.85 (1.57-2.24)	1.67 (1.52-1.88)	1.71 (1.52-1.75)
MELD score	18.79 (15.36-22.47)	23.51 (20.75-25.10) * ^b^	17.92 (16.41-19.51)	22.38 (19.91-24.23) *** ^b^	16.53 (13.28-20.86)	17.65 (16.45-20.56)	15.16 (12.84-19.61)	14.04 (10.37-15.99)

Quantitative variables were expressed as the means±SD or the median (centile 25; centile 75). Categorical variables were expressed as number (%).

*p<0.05, **p<0.01, ***p<0.001 and ****p<0.0001 for comparisons between survivors and non-survivors group. a unpaired t test. b Mann–Whitney U test.

SMT, standard medical treatment; SMT+GC, standard medical treatment plus glucocorticoid; ACHBLF, acute-on-chronic hepatitis B liver failure; CHB, chronic hepatitis B; HC, healthy controls; HBeAg, hepatitis B virus e antigen; HBV, hepatitis B virus; ALT, alanine aminotransferase; AST, aspartate transaminase; ALB, Albumin; TBiL, total bilirubin; Cr, creatinine; BUN, blood urea nitrogen, PTA, prothrombin activityprothrombin time activity; INR, international normalized ratio; MELD, model for end-stage liver disease.

### IL-6 and SOCS3 mRNA levels of ACHBLF and pre-ACHBLF patients with different treatment and different 90d prognosis

**Figure 4:**
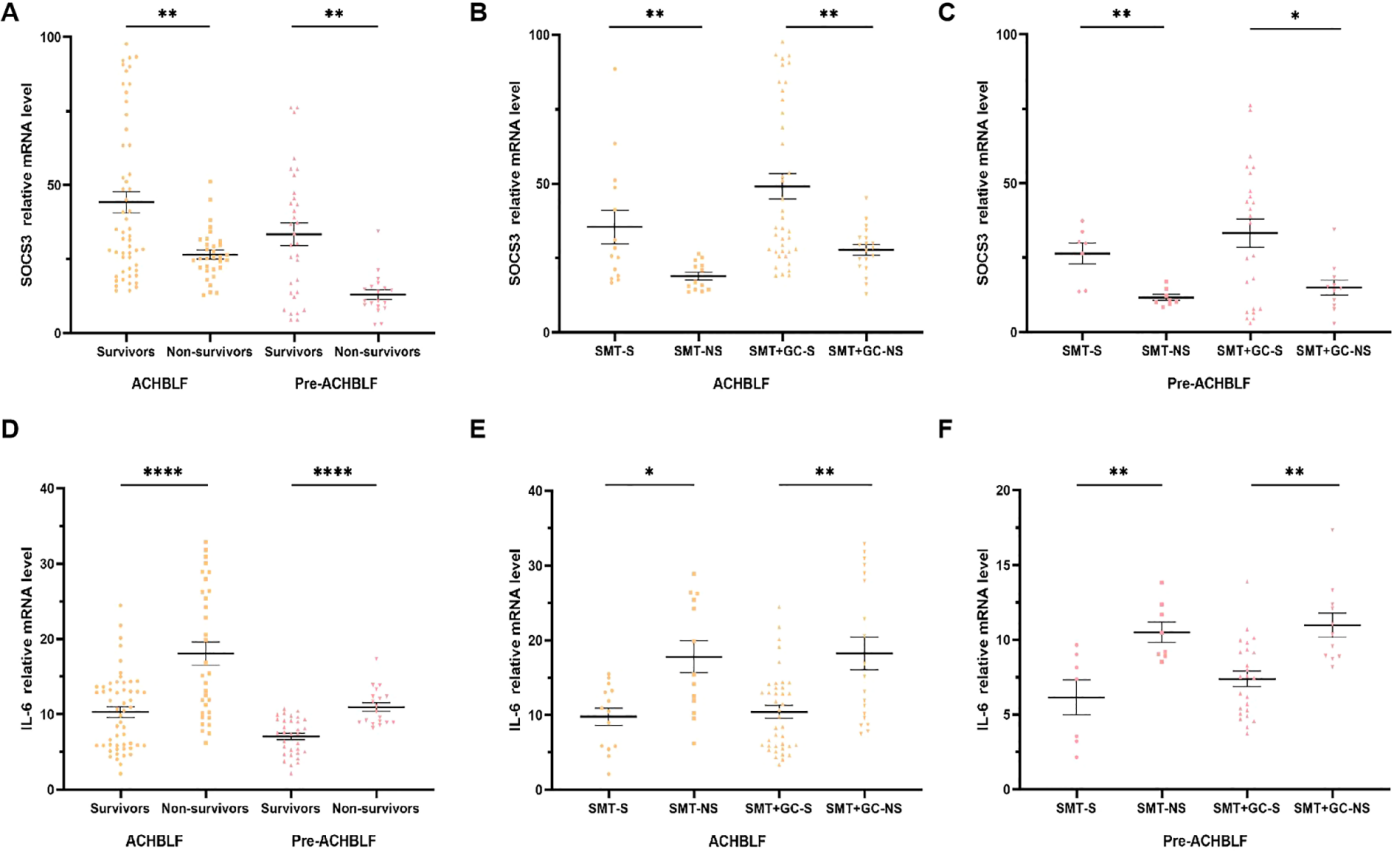
SOCS3 and IL-6 mRNA levels of ACHBLF and pre-ACHBLF patients with different treatment and different 90d prognosis. **(A)** SOCS3 mRNA levels of non-survivors in ACHBLF and pre-ACHBLF groups were significantly lower than that of survivors. **(B)** SOCS3 mRNA levels of non-survivors in ACHBLF different treatment groups were significantly lower than that of survivors. **(C)** SOCS3 mRNA levels of non-survivors in pre-ACHBLF different treatment groups were significantly lower than that of survivors. **(D)** IL-6 mRNA levels of non-survivors in ACHBLF and pre-ACHBLF groups were significantly higher than that of survivors. **(E)** IL-6 mRNA levels of non-survivors in ACHBLF different treatment groups were significantly higher than that of survivors. **(F)** IL-6 mRNA levels of non-survivors in pre-ACHBLF different treatment groups were significantly higher than that of survivors. * p<0.05; ** p<0.01; ****p<0.0001.

As illustrated in **Figures 4A, B**, for 90-day prognosis, SOCS3 mRNA levels were significantly higher in survivors of ACHBLF or pre-ACHBLF patients (n=53/31, 32.87[21.51-66.20]/30.38[13.62-45.57]) than in non-survivors (n=32/19, 25.46[21.72-30.81]/10.78[9.01-15.67], *p*=0.0084/0.0016). In the SMT group of ACHBLF or pre-ACHBLF patients, SOCS3 mRNA levels were significantly higher in survivors (n=14/7, 27.03[18.83-49.32]/29.82[13.88-33.75]) than in non-survivors (n=13/8, 16.92[14.36-23.44]/10.52[9.63-13.94], *p*=0.0034/0.0037) (**Figures 4B, C**). In addition, in the SMT+GC group of ACHBLF patients, SOCS3 mRNA levels were significantly higher in survivors (n=39, 35.26[27.12-78.21]) than in non-survivors (n=19, 28.34[22.18-31.89]) (*p*=0.0062), as well as in pre-ACHBLF patients (n=24/11, 37.95[7.87-51.94] vs. 14.81[9.01-18.40]) (*p*=0.0467) (**Figures 4B, C**).

IL-6 mRNA levels were significantly higher in survivors of ACHBLF or pre-ACHBLF patients (n=53/31, 10.49[5.83-13.66]/7.47[4.88-9.17]) than in non-survivors (n=32/19, 15.27[10.20-26.36]/10.35[8.91-12.35], *p*<0.0001/<0.0001) (**Figure 4D**). In the SMT group of ACHBLF or pre-ACHBLF patients, IL-6 mRNA levels were significantly higher in survivors (n=14/7, 10.71[5.73-13.48]/7.37[3.22-9.03]) than in non-survivors (n=13/8, 15.41[11.09-25.83]/9.83[8.95-12.19], *p*=0.0107/0.0093) (**Figures 4E, F**). In the SMT+GC group of ACHBLF or pre-ACHBLF patients, IL-6 mRNA levels were significantly higher in survivors (n=39/24, 9.84[5.83-13.66]/7.50[5.11-9.30]) than in non-survivors (n = 19/11, 15.14[9.61-28.94]/10.35[8.89-12.35], *p*=0.0013/0.0012) (**Figures 4E, F**).

### General characteristics of patients receiving glucocorticoid therapy in different 90-day prognosis

**Table 5** showed general characteristics of patients who received glucocorticoid therapy in different 90-day prognosis. BUN in non-survivors (n=30, 6.15[4.22-8.73]) were significantly higher than in survivors (n=63, 4.40[3.35-5.95]) (*p*=0.003). INR in non-survivors (n=30, 1.83[1.71-2.40]) were significantly higher than in survivors (n=63, 1.73[1.55-2.09]) (*p*=0.015). MELD score in non-survivors (n=30, 19.95[14.85-23.26]) were significantly higher than in survivors (n=63, 17.35[14.69-19.51]) (*p*=0.045).

**Table 5:** General characteristics of patients receiving glucocorticoid therapy with different 90-day prognosis.

Variable	patients receiving glucocorticoid therapy (n=93)		P value
	Survivors (n=63)	Non-survivors (n=30)	
Age (years)	49.00 (40.00-58.00)	51.00 (47.25-57.50)	0.435 ^b^
Gender (male,%)	45 (71.43)	20 (66.67)	0.640 ^b^
HBeAg positive (n,%)	40 (63.49)	15 (50.00)	0.216 ^b^
HBV DNA (log10 IU/ml)	3.65 (2.81-5.04)	3.66 (2.65-5.16)	0.802 ^b^
ALT (U/L)	176.00 (127.50-258.00)	182.00 (151.75-266.50)	0.590 ^b^
AST (U/L)	160.00 (122.50-234.50)	157.00 (125.25-224.50)	0.824 ^b^
ALB (g/L)	32.90 (29.15-35.55)	31.30 (29.42-35.25)	0.370 ^b^
TBiL (μmol/L)	193.70 (135.40-305.00)	222.65 (144.98-287.45)	0.724 ^b^
Cr (μmol/L)	53.00 (43.50-62.50)	55.50 (42.00-72.00)	0.678 ^b^
BUN (mmol/L)	4.40 (3.35-5.95)	6.15 (4.22-8.73)	0.003 ^b^
PTA	43.00 (35.00-47.00)	41.00 (29.25-46.00)	0.106 ^b^
INR	1.73 (1.55-2.09)	1.83 (1.71-2.40)	0.015 ^b^
MELD score	17.35 (14.69-19.51)	19.95 (14.85-23.26)	0.045 ^b^

Quantitative variables were expressed as the means±SD or the median (centile 25; centile 75). Categorical variables were expressed as number (%).

a unpaired t test. b Mann–Whitney U test.

HBeAg, hepatitis B virus e antigen; HBV, hepatitis B virus; ALT, alanine aminotransferase; AST, aspartate transaminase; ALB, Albumin; TBiL, total bilirubin; Cr, creatinine; BUN, blood urea nitrogen, PTA, prothrombin activityprothrombin time activity; INR, international normalized ratio; MELD, model for end-stage liver disease.

### SOCS3 and IL-6 mRNA levels of patients receiving glucocorticoid therapy in different 90-day prognosis

**Figure 5:**
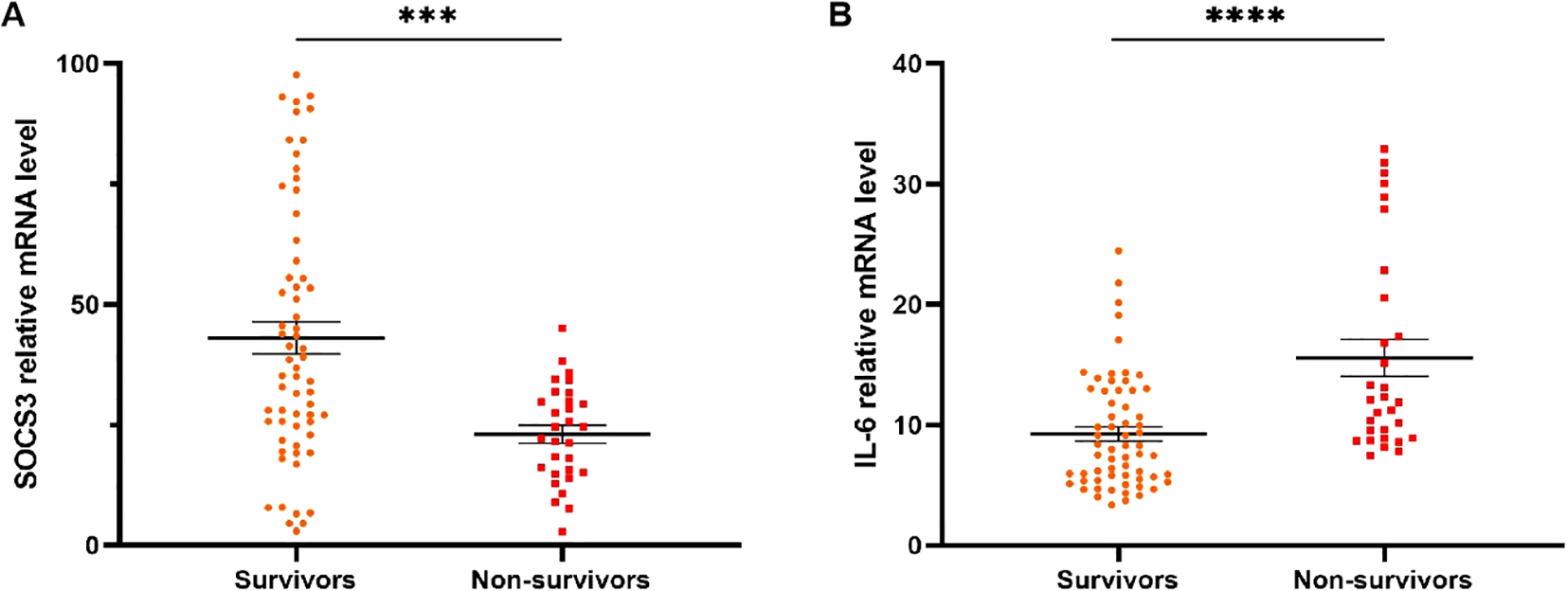
SOCS3 and IL-6 mRNA levels of patients receiving glucocorticoid treatment with different 90d prognosis. **(A)** SOCS3 mRNA levels of non-survivors in patients receiving glucocorticoid treatment were significantly lower than that of survivors. **(B)** IL-6 mRNA levels of non-survivors in patients receiving glucocorticoid treatment were significantly higher than that of survivors. *** p<0.001; ****p<0 .0001.

**Figures 5A, B** presented SOCS3 and IL-6 mRNA levels of patients receiving glucocorticoid therapy in different 90-day prognosis. For 90-day mortality, SOCS3 mRNA levels were significantly higher in survivors (n=63, 36.80[24.79-59.06]) than in non-survivors (n=30, 23.38[15.07-30.33], *p*=0.0002) (**Figure 5A**). IL-6 mRNA levels were also significantly higher in non-survivors (n=30, 11.99[8.91-21.11]) than in survivors (n=63, 7.99[5.50-12.88], *p*<0.0001) (**Figure 5B**).

### Prediction value of IL-6 and SOCS3 mRNA levels for 90-day prognosis of patients receiving glucocorticoid therapy

Based on the univariate and multivariate logistic regression analysis results, SOCS3 relative mRNA level (OR=1.240, 95%CI: 1.007-1.528, *p*=0.043), IL-6 relative mRNA level (OR=0.932, 95%CI: 0.889-0.977, *p*=0.003), and BUN (OR=1.210, 95%CI: 1.071-1.366, *p*=0.002) were significantly associated with 90d prognosis of ACHBLF and pre-ACHBLF patients receiving glucocorticoid therapy (**Table 6**).

**Table 6:** Univariate and multivariate logistic regression analysis of predictors associated with 90-day prognosis in patients receiving glucocorticoid therapy.

Variables	Univariate logistic regression analysis			Multivariate logistic regression analysis		
	β	OR (95%CI)	P value	β	OR (95%CI)	P value
HBV DNA	-0.020	0.980 (0.754,1.273)	0.880			
ALT	-0.000	1.000 (0.997,1.002)	0.985			
AST	-0.001	0.999 (0.996,1.003)	0.795			
ALB	-0.039	0.962 (0.879,1.052)	0.394			
TBiL	0.000	1.000 (0.997,1.004)	0.930			
Cr	0.014	1.014 (0.995,1.034)	0.138			
BUN	0.235	1.265 (1.076,1.486)	0.004	0.215	1.240 (1.007,1.528)	0.043
PTA	-0.035	0.965 (0.924,1.009)	0.119			
INR	1.193	3.298 (1.332,8.165)	0.010			
SOCS3 relative mRNA level	-0.053	0.948 (0.919,0.979)	0.001	-0.070	0.932 (0.889,0.977)	0.003
IL-6 relative mRNA level	0.153	1.165 (1.071,1.267)	0.000	0.190	1.210 (1.071,1.366)	0.002

β, partial regression coefficient; SE, Standard error; OR, odds ratio; CI, confidence interval.

HBV, hepatitis B virus; ALT, alanine aminotransferase; AST, aspartate transaminase; ALB, Albumin; TBiL, total bilirubin; Cr, creatinine; BUN, blood urea nitrogen, PTA, prothrombin activityprothrombin time activity; INR, international normalized ratio; SOCS, suppressor of cytokine signaling; IL, interleukin.

**Figure 6:**
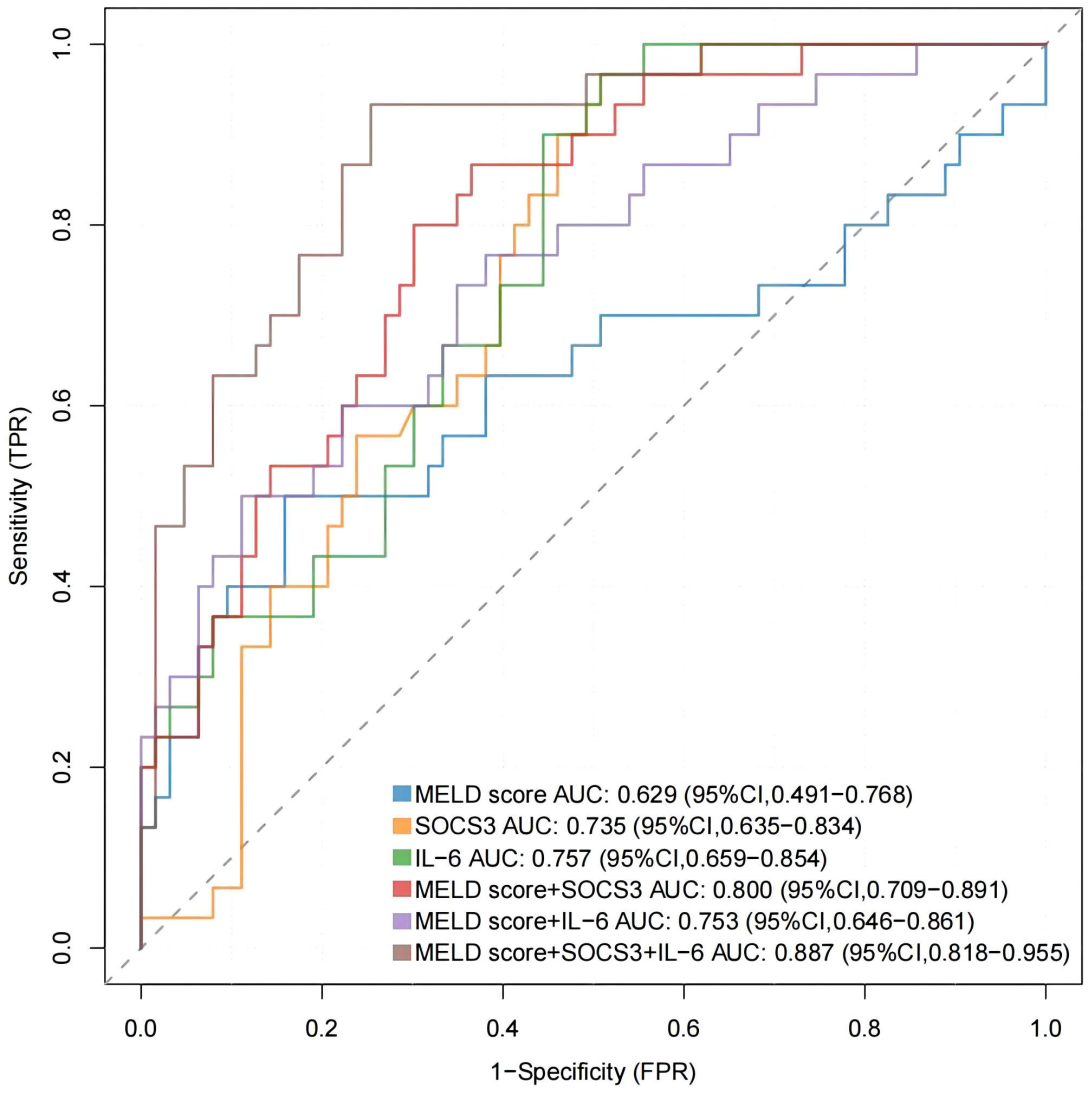
The ROC curves were drawn to evaluate the discrimination of MELD score, SOCS3 and IL-6 mRNA levels.

MELD score is a common score to evaluate the prognosis of ACHBLF patients, and the combination of SOCS3 and IL-6 may more accurately predict the prognosis. As shown in **Figure 6**, receiver operating characteristic (ROC) curves were drawn and the area under the curve (AUC) was calculated to evaluate the discrimination of the indicators. The AUC of MELD score was 0.629 (95% CI 0.491-0.768) and the optimal cut-off value was 20.64, with sensitivity of 50.0%, specificity of 84.1%. The AUC of SOCS3 was 0.735 (95%CI 0.635-0.834) and the AUC of IL-6 was 0.757 (95%CI 0.659-0.854). Their optimal cut-off value was 7.23/7.70, with sensitivity of 96.7%/96.7%, specificity of 7.9%/49.2%. The AUC of MELD score+SOCS3 was 0.800 (95%CI 0.709-0.891) and the AUC of MELD score+IL-6 was 0.753 (95%CI 0.646-0.861). Their optimal cut-off value was 0.28/0.40, with sensitivity of 86.7%/50.0%, specificity of 63.50%/88.90%. The AUC of MELD score+SOCS3+IL-6 was 0.887 (95%CI 0.818-0.955). The optimal cut-off value was 0.29, with sensitivity of 93.3%, specificity of 74.6%. The formula of MELD score+SOCS3+IL-6 was -2.807 + 0.124*MELD score -0.083*SOCS3 relative mRNA expression +0.189*IL-6 relative mRNA expression.

### Effect of glucocorticoid treatment on the short-term prognosis of ACHBLF and pre-ACHBLF patients

**Figure 7:**
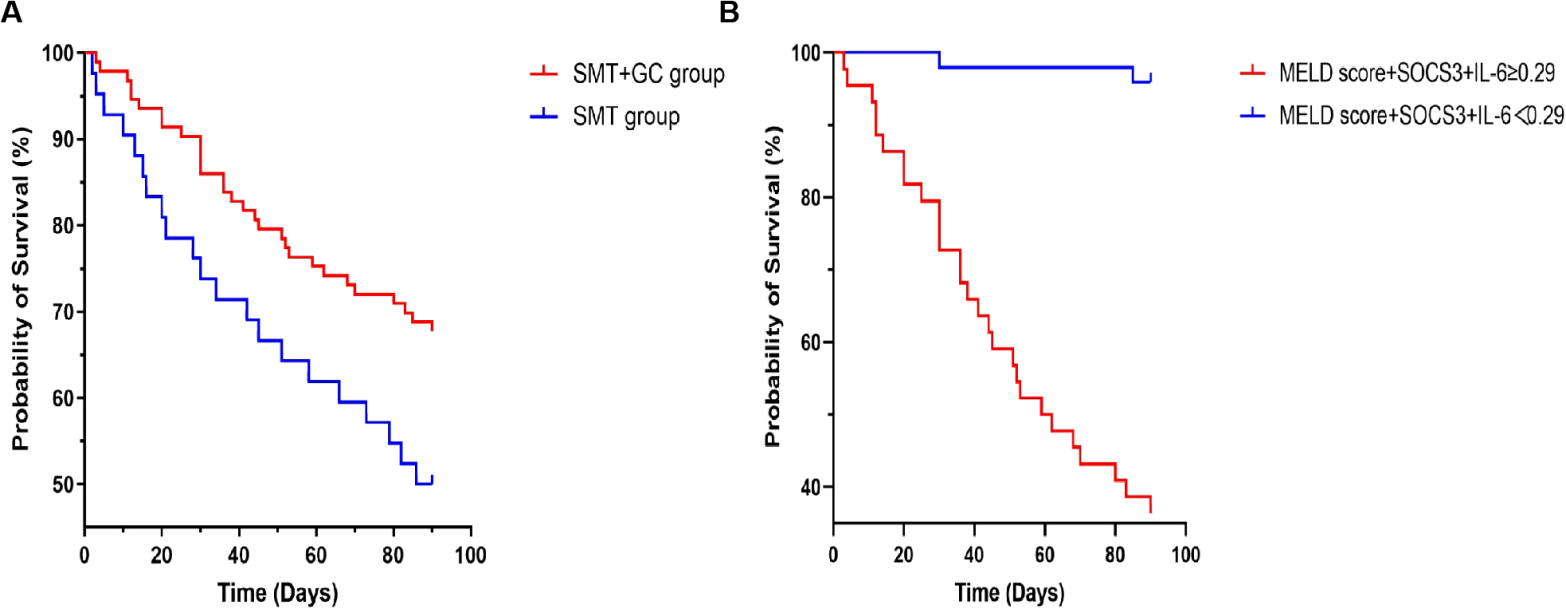
Kaplan–Meier curves for glucocorticoid (GC) and standard medical treatment (SMT) therapy. **(A)** Glucocorticoid therapy reduces mortality in ACHBLF and pre-ACHBLF patients. **(B)** The optimal cut-off value of MELD score+SOCS3+IL-6 at 0.29 identifies the risk of death in ACHBLF patients with glucocorticoid treatment.

Kaplan-Meier curves were drawn to further monitor the effect of glucocorticoid treatment on the short-term prognosis of patients enrolled in this study. As shown in **Figure 7A**, using 90-day prognosis as the cut-off point, survival rate of the SMT+GC group was significantly higher than that of the SMT group (log-rank test with *p*=0.0350). Taking the optimal cut-off value of 0.29 for MELD score+SOCS3+IL-6 as the boundary, survival rates of SMT+GC group with a score higher than 0.29 were significantly lower than those with a score lower than 0.29 (log-rank test with *p*<0.0001) (**Figure 7B**).

## Discussion

IL-6 is an important cytokine involved in cytokine storm. The mechanism of ACHBLF is still unclear, but immune imbalance and cytokine storm are closely related to the pathogenesis of ACHBLF. The excessive immune response in ACHBLF patients leads to excessive cytokines release, resulting in cytokine storm. IL-6, TNF-α, IL-10 and other cytokines are important participating factors in the infection related cytokine storm (Fajgenbaum and June, 2020). Glucocorticoids possess powerful anti-inflammatory properties and have been applied in clinical treatment of ACHBLF (Scherholz et al., 2019; Jia et al., 2020). Glucocorticoids can affect the cytokine levels during the anti-inflammatory treatment, such as IL-6, IL-1β, TNF-α, etc (Barnes, 1998). A previous study have employed Luminex assay to measure the concentrations of 40 kinds of composite cytokines and chemokines in ACHBLF patients and the results revealed that the expressions of some cytokines varied among patients with different prognosis (Zhu et al., 2023). In the present study, we measured serum cytokines and found that IL-6, IL-1β, IL-18 and TNF-α levels were significantly higher in the non-survivors of patients receiving glucocorticoid treatment, while IL-10 level was significantly lower. Present results of serum cytokine detection are consistent with that of previous studies. We further analyzed the importance of serum cytokines in the prognosis of patients receiving glucocorticoid treatment and found that IL-6 ranked first. IL-6 plays a crucial role in host defense against infection and tissue injuries and is a bioindicator of multiple distinct types of cytokine storm (Kang and Kishimoto, 2021). A previous study reported that a high level of serum IL-6 was an independent risk factor for mortality in ACHBLF patients and a sustained high level or dynamic elevated level of serum IL-6 indicated a higher mortality (Zhou et al., 2020).

In ACHBLF patients, the excessive activation of both innate and adaptive immunity leads to the production of a substantial amount of inflammatory cytokines. Specifically, the peripheral blood mononuclear macrophage system releases significant quantities of cytokines, such as IL-6, into the peripheral blood (Br and Sarin, 2023). Furthermore, peripheral blood mononuclear cells (PBMCs) are regarded as the first line of defense of the immune system against inflammation, and previous studies have reported that host immune or inflammatory responses can influence gene expression of PBMC in various diseases (Ma et al., 2015; Haß et al., 2023). Previous studies have reported PBMCs were used to seek biomarkers for predicting ACHBLF, which could reflect the immune and inflammatory status (Li et al., 2022; Hassan et al., 2024). In this study, we report that IL-6 mRNA levels in PBMCs were significantly increased in ACHBLF patients. IL-6 mRNA levels were significantly higher in non-survivors of ACHBLF and pre-ACHBLF patients. In patients receiving glucocorticoid treatment, IL-6 mRNA levels in non-survivors than that of survivors.

In addition, the signaling pathway of cytokines is mainly regulated through the JAK/STAT pathway and the SOCS protein family has a role in attenuating this pathway. SOCS3 is the primary inhibitor of IL-6 family of cytokines signaling, interacting with gp130 and JAK1, JAK2 and TYK2 to control both the duration of signaling and the biological response (Babon et al., 2014). The results of one previous study showed that SOCS3 expression was increased in liver tissues and PBMCs of ACHBLF patients and increased in livers of BALB/cJ mice 72 hours after infection (Li et al., 2014). Based on the above study results, SOCS3 may be correlated with the prognosis of ACHBLF patients receiving glucocorticoid treatment. We also found that SOCS3 mRNA levels in PBMCs were increased in ACHBLF patients, and was positively correlated with IL-6 mRNA levels. Furthermore, we reported that SOCS3 mRNA levels was significantly decreased in non-survivors of ACHBLF and pre-ACHBLF patients. In ACHBLF and pre-ACHBLF patients receiving glucocorticoid therapy, SOCS3 mRNA expression was significantly decreased in non-survivors.

ACHBLF is characterized by acute onset, rapid development and high mortality and thus early and effective treatment for ACHBLF and pre-ACHBLF is particularly important. Glucocorticoids can rapidly inhibit excessive immune response and inflammatory response. In a previous case report, a combination of entecavir and early-phase corticosteroid was effective in the treatment of severe exacerbation of chronic hepatitis B (Matsumoto et al., 2009). The results of another clinical trial showed that the introduction of high-dose corticosteroid could reverse deterioration in patients with early stage of severe exacerbation of chronic hepatitis B (Fujiwara et al., 2005). Based on above studies, this is a reasonable treatment option to use glucocorticoid, which can suppress the excessive immune response and inflammatory response in ACHBLF patients. However, the glucocorticoids use remains controversial and only a part of ACHBLF patients can benefit from glucocorticoid treatment. It is an urgent need to explore predictors for ACHBLF patients treated with glucocorticoid. The prognosis prediction for ACHBLF patients has always been a key point in the research on acute exacerbation of chronic hepatitis. MELD score is commonly used to predict for ACHBLF patients, but it still has limitations (Kamath et al., 2007).

The univariate and multivariate logistic regression analysis results in this study showed that SOCS3 and IL-6 relative mRNA levels were significantly associated with 90-day prognosis of ACHBLF and pre-ACHBLF patients receiving glucocorticoid therapy. We reported that the AUC of MELD score combined with SOCS3 and IL-6 in predicting the prognosis of ACHBLF patients receiving glucocorticoid therapy was 0.887 (95%CI 0.818-0.955) and the optimal cut-off value was 0.29, with sensitivity of 93.3%, specificity of 74.6%, which indicated SOCS3 and IL-6 mRNA levels may serve as predictors for ACHBLF patients receiving glucocorticoid therapy and improve the predictive power of MELD score. Finally, by drawing Kaplan-Meier curves, it was found that glucocorticoid treatment could improve survival rates of patients, and MELD score+SOCS3+IL-6 could be used to predict for patients receiving glucocorticoid treatment.

When glucocorticoid therapy was gradually applied to the clinical treatment of ACHBLF, more and more studies began to explore more biomarkers and models to effectively predict for ACHBLF patients receiving glucocorticoid treatment. Our previous study proposed a HITAS score, an early prediction model for the prognosis of ACHBLF, which might be used to identify ACHBLF patients with favorable responses to glucocorticoid treatment (Gao et al., 2022). Zhang et al. demonstrated that ACHBLF patients without SOCS1 promoter methylation may have a favorable response to corticosteroid treatment (Zhang et al., 2014). Wang et al. showed that ACHBLF patients with low levels of thymosin β4 methylation may show a more favorable response to glucocorticoid treatment (Wang et al., 2023). Not only ACHBLF, but a previous study sought early predictors that could accurately identify non-response to steroids in patients with ACLF due to autoimmune hepatitis (AIH) (Sharma et al., 2023).

The pathogenesis of ACHBLF and the mechanism of glucocorticoid treatment are closely associated with inflammation. Our present study revealed that ACHBLF and pre-ACHBLF non-survivors receiving glucocorticoid therapy had lower SOCS3 mRNA levels and higher IL-6 mRNA levels. The results suggest that ACHBLF patients who have no response to glucocorticoid therapy may have more severe inflammatory response and weaker anti-inflammatory ability. Meanwhile, we showed that SOCS3 and IL-6 had a potential role in the prediction of patients. Although the MELD score is most widely used to predict the prognosis of patients with end-stage liver disease, approximately 15%-20% of patients whose survival cannot be accurately predicted by the MELD score (Kamath et al., 2007). SOCS3 and IL-6 relative mRNA levels were combined with the MELD score, which could predict for ACHBLF patients treated with glucocorticoid more accurately.

However, there are some limitations of this study. Firstly, SOCS3 and IL-6 mRNA expression levels in the liver tissues of patients were not analyzed because of the higher risk of liver biopsy in ACHBLF patients. Second, the sample size was relatively small, and all patients were in a single center. In the future, multi-center, large-sample, and prospective follow-up studies are needed to further confirm the results of this study. Finally, IL-6, as the most common pro-inflammatory cytokine, the precise and special molecular mechanism about how IL-6 and SOCS3 were involved in the progress of ACHBLF remained unclear and might also be studied in further study.

In conclusion, this study provides new evidence for the feasibility of a clinical regimen of glucocorticoid therapy to improve survival rates in AHCBLF. The serum cytokine levels in ACHBLF patients receiving glucocorticoid therapy with different prognoses had significant differences. IL-6 plays an important role in influencing the prognosis of patients receiving glucocorticoid therapy. SOCS3 and IL-6 mRNA could be used as a potential biomarker to predict for ACHBLF patients receiving glucocorticoid therapy. SOCS3 and IL-6 relative mRNA levels combined with the MELD score could improve the ability to early predict for ACHBLF patients receiving glucocorticoid therapy.

## Conclusion

Glucocorticoid therapy is an effective strategy for ACHBLF patients, increasing the 90-day survival rates. IL-6 plays an important role in influencing the prognosis of patients receiving glucocorticoid therapy. SOCS3 and IL-6 mRNA levels in PBMC improve the predictive ability for ACHBLF patients receiving glucocorticoid therapy. SOCS3+IL-6+MELD score identifies the risk of death in ACHBLF patients with glucocorticoid treatment.

## Data Availability

The datasets presented in this study can be found in online repositories. The names of the repository/repositories and accession number(s) can be found in the article/supplementary material.
